# Cyclodextrin-Based Delivery Systems for Flavonoids: Mechanisms, Advances, Formulation, and Application Opportunities

**DOI:** 10.3390/antiox14080998

**Published:** 2025-08-14

**Authors:** Ferenc Fenyvesi, Ágnes Klusóczki, Ágnes Rusznyák, Barbara Zsebik, Ildikó Bácskay, Judit Váradi

**Affiliations:** 1 Department of Molecular and Nanopharmaceutics, Faculty of Pharmacy, University of Debrecen, H-4002 Debrecen, Hungary; fenyvesi.ferenc@pharm.unideb.hu (F.F.); rusznyak.agnes@euipar.unideb.hu (Á.R.); 2 Department of Pharmaceutical Technology, Faculty of Pharmacy, University of Debrecen, H-4002 Debrecen, Hungary; klusoczki.agnes@pharm.unideb.hu (Á.K.); bacskay.ildiko@pharm.unideb.hu (I.B.); 3 Department of Biopharmacy, Faculty of Pharmacy, University of Debrecen, H-4002 Debrecen, Hungary; zsebik.barbara@pharm.unideb.hu

**Keywords:** flavonoids, cyclodextrin, antioxidant, flavonoid–cyclodextrin complexes

## Abstract

Flavonoids play an important role in preventive and therapeutic research due to their significant antioxidant properties. However, their application is limited by several pharmacokinetic drawbacks, such as poor water solubility and low bioavailability. Cyclodextrin-based delivery systems offer an opportunity to overcome these disadvantages. Cyclodextrins are able to form stable, water-soluble inclusion complexes with flavonoids, thereby improving their solubility, chemical stability, and antioxidant activity. This review summarizes the structural characteristics and complexation mechanisms of various flavonoid–cyclodextrin complexes and examines how these interactions influence biological activity. Special attention is given to nanotechnological formulations—such as liposomes, nanofibers, and nanosponges—that enable targeted drug delivery and enhanced therapeutic efficacy. The aim of this review is to provide a comprehensive overview of the role of cyclodextrin-based carriers in the formulation of flavonoids and to highlight the future potential of these systems in modern therapeutics and functional product development.

## 1. Antioxidant Properties of Flavonoids

Flavonoids play a significant role in maintaining human health due to their special biological activity. Through their antioxidant effect, flavonoids can reduce the level of free radicals and reactive oxygen species (ROS), which are the cause of many diseases, such as cancer, cardiovascular problems, and aging. Their antioxidant properties can be achieved through different mechanisms [1].

Flavonoids react directly with free radicals, neutralizing them. Flavonoid molecules are able to donate electrons to free radicals, thus preventing their harmful effects, which can otherwise lead to damage to cell structures and DNA [2].

Flavonoids can bind metal ions, such as iron or copper, in the form of chelate complexes, thus inhibiting the formation of free radicals, which are formed by the direct action of metals. This mechanism is particularly important because metals can catalyze oxidative stress and promote inflammation [3].

In addition, they also affect the function of antioxidant enzymes. For example, they can activate glutathione peroxidase and superoxide dismutase, which play a key role in protecting cells against oxidative stress [2]. Flavonoids can reduce inflammatory processes and help protect cells during inflammatory responses. This is especially important in chronic diseases such as vascular diseases or diabetes [4,5].

Although flavonoids are extremely promising for health maintenance and disease prevention, their use has several limiting factors. One of the major limiting factors is the low bioavailability of flavonoids. Since flavonoids are often large molecules and poorly soluble in water, their absorption and metabolism in the body are not always optimal. Their rapid degradation in the digestive tract and metabolism in the liver can significantly reduce their effectiveness [6].

They can interact with many drugs and other compounds in the body, which can affect their effectiveness. For example, some flavonoids can reduce the absorption of drugs or increase their toxic effects by affecting the activity of enzymes that metabolize drugs. Proper dosing of flavonoids can also be difficult, as the effects of individual flavonoids depend significantly on the amount consumed and their bioactive form [7].

Given all this, the development of flavonoid-containing formulations aims to increase bioavailability and enhance stability. The former challenges can be met using cyclodextrins. These carbohydrate-based carrier systems, due to their structural variability, offer the opportunity to improve the applicability of the entire flavonoid group.

## 2. Cyclodextrin Chemistry

**Figure 1:**
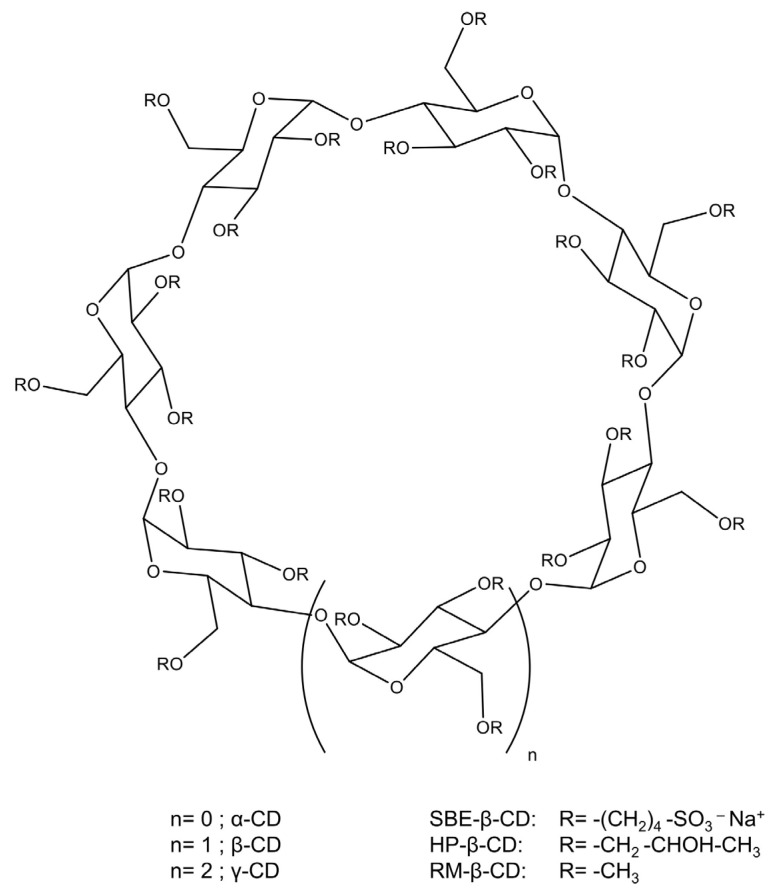
Structure of cyclodextrin derivatives.

Cyclodextrins are a special class of non-reducing cyclic oligosaccharides composed of glucopyranose units. These glucose molecules are linked in such a way that they form a conical cylinder with a hydrophobic inner cavity and a hydrophilic outer surface (Figure 1). The three main types are the α-, β-, and γ-cyclodextrins, containing 6, 7, and 8 glucopyranose units, respectively, and are most commonly used for the complexation of molecules, which are less polar than water [8,9]. The resulting noncovalent complexes improve the physicochemical properties of the guest molecule, including its aqueous solubility and stability, and at the same time, the bioavailability of the guest from the complex is also improved [9,10]. For successful host–guest complexation, the guest molecule must fit appropriately into the cyclodextrin cavity, making its size and molecular structure critical factors. Parent cyclodextrins (α-, β-, and γ-CD) expose hydroxyl groups on their exterior surface and are water-soluble; however, interestingly, β-CD is less soluble than the others [11,12]. The properties of the parent cyclodextrins were further enhanced through [9]. These derivatives exhibit significantly improved solubility and complexation abilities, while also demonstrating reduced toxicity in the case of hydroxypropyl and sulfobutyl derivatives of the β-cyclodextrin [13]. The cyclodextrin rings can be substituted with various substituents; the most commonly used derivatives include methyl, hydroxypropyl, and sulfobutyl substituents, which are covalently attached to the cyclodextrin ring. In fact, beyond the parent cyclodextrins, hydroxypropyl and sulfobutyl derivatives of the β-cyclodextrin are officially recognized by both the European Pharmacopoeia (Ph. Eur.) and the United States Pharmacopeia (USP) and are primarily employed as solubility and bioavailability enhancers in pharmaceutical formulations [14].

**Figure 2:**
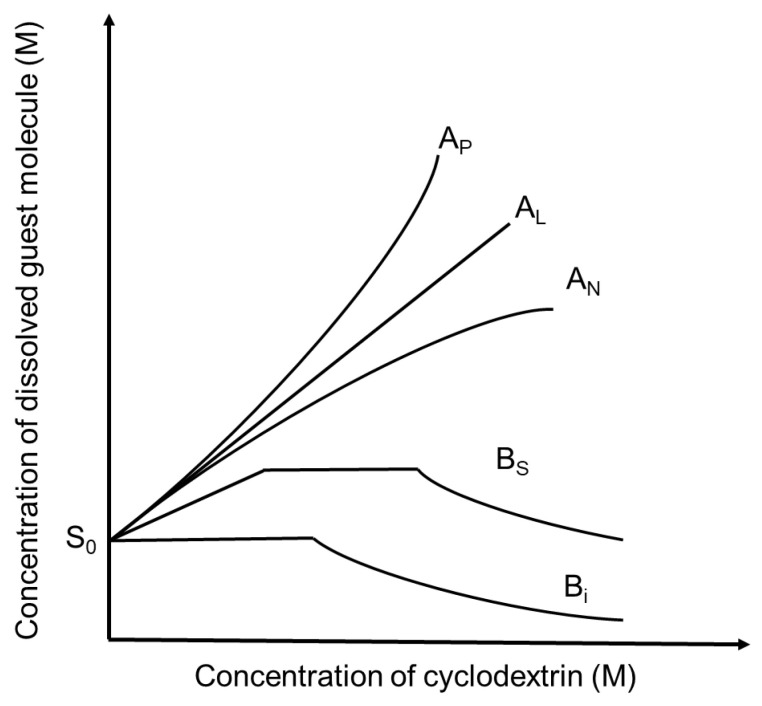
Representative phase-solubility diagrams of guest–cyclodextrin systems according to the Higuchi–Connors classification. A_L_-type curves indicate a linear relationship, A_P_-type curves show positive deviation, and A_N_-type curves exhibit negative deviation. B-type curves (B_S_ and B_i_ subtypes) represent systems where the complex has limited or lower solubility than the free guest molecule. S_0_ is the intrinsic solubility of the free guest molecule in aqueous medium in the absence of cyclodextrin.

The complexation of cyclodextrins can be monitored through the solubilization of guest molecules; the concentration of the guest increases significantly in the dissolved phase [8]. In solution, increasing the concentration of cyclodextrin typically leads to a corresponding increase in the concentration of solubilized guest molecules. This relationship is often illustrated using phase-solubility diagrams, which plot the concentration of the solubilized guest compound as a function of increasing cyclodextrin concentration in aqueous solution [15]. However, the behavior can sometimes be more complex than linear (see Figure 2).

In an A_L_-type phase solubility diagram, where a linear increase in the solubility of the guest is observed with increasing cyclodextrin (CD) concentration, the complex is typically first-order with respect to CD and first-order with respect to the drug, indicating a 1:1 stoichiometry. However, in some cases, higher-order complexes (e.g., 1:2 guest:CD) may form, which would still appear linear under certain conditions. If the curve shows a positive deviation from linearity (A_P_-type), it indicates a higher-order complex (e.g., 1:2 or 1:n) with respect to CD, or molecular aggregation that enhances solubilization. Conversely, a negatively deviating curve (A_N_-type) indicates that the solubility of the guest or the complex decreases at higher cyclodextrin concentrations due to the self-association of CD molecules or their complexes. In some cases, B-type curves may appear, where the formed complexes have limited or even lower solubility than the free guest molecule, indicating poor complexation efficiency or insoluble complex formation [9].

Using an A_L_-type phase-solubility diagram, the stability constant (K_1:1_) of a 1:1 guest–cyclodextrin complex can be calculated using the following equation:

$$K_{1:1}=\frac{slope}{S_{0}(1-slope)}$$

The binding constants of drug–cyclodextrin complexes typically range between 10 and 2000 M^−1^ but are generally lower than 5000 M^−1^. This range is considered optimal for enhancing the bioavailability of drugs or biologically active molecules [9,16]

There is no evidence that cyclodextrins have a direct antioxidant effect or directly scavenge free radicals. However, cyclodextrins can act as secondary antioxidants through complexation, solubility enhancement, stabilization, and protection of antioxidant compounds. Antioxidants with different chemical structures can be protected by cyclodextrins. For example, the oxidation rate of edaravone was controlled by α- and β-cyclodextrin, while interestingly, 2-hydroxypropyl-β-cyclodextrin preserved the antioxidant capacity of a system containing ascorbic acid [17,18].

It should also be mentioned that cyclodextrins are increasingly used as active ingredients for stabilizing and removing bioactive compounds and for indirectly modulating oxidative stress. Two notable examples with therapeutic relevance are based on the complexation and removal of oxidized metabolites from tissues. Firstly, sulfobutylether-β-cyclodextrin is used as an orphan drug for the treatment of Stargardt disease. It reduces the level of accumulated toxic bisretinoids, which generate ROS and are implicated in retinal diseases. Secondly, a specifically designed β-cyclodextrin dimer can remove the accumulated toxic oxidized cholesterol, 7-ketocholesterol, from atherosclerotic plaques [19]. Antioxidant flavonoids have a preferred chemical structure for complexation with cyclodextrins, offering broader implications. This review further explores the application of cyclodextrins in flavonoid complexation.

## 3. Flavonoids

### 3.1. Structural Properties of Flavonoids

**Figure 3:**
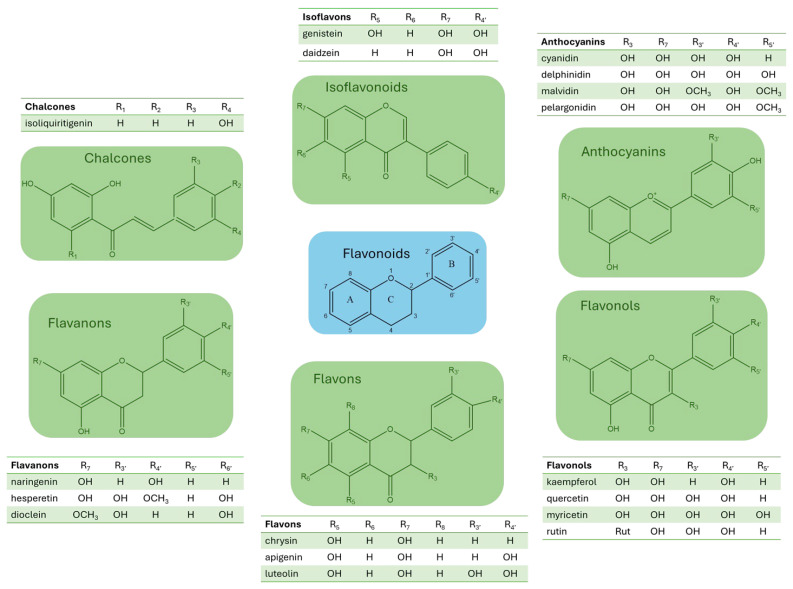
Structure of flavonoid subclasses and substituents of some representative derivatives.

Flavonoids can be structurally classified based on their heterocyclic ring system and the connection between the benzene and benzopyran rings into the following groups: flavones, flavonols, flavanones, flavanonols, flavanols, flavan-3-ols, anthocyanidins, and isoflavonoids. The various flavonoid compounds differ in their oxidation states and in the substitution pattern of the C ring (see Figure 3). Each group of flavonoids possesses a distinct and specific set of substituents, which are attached to different carbon atoms. The most common substituents include -H, -OH, -O/C-glycoside, -mannoside, -galactoside, -methyl, -gallate, and -acyl groups [20]. Most substituents are linked to the core flavonoid scaffold found in plants, which in many cases stabilizes the molecular structure. In fruits, the most stable form of flavonoids is the flavonoid-*O*-glycoside, making these the most common flavonoids in nature. Glycosylation sites result in great diversity, and both *O*-glycosides and *C*-glycosides are distinguished. Both types of glycosidic bonds can be observed simultaneously on an aglycone. The sugar substituent of flavonoid glycosides is most often composed of d-apose, d-arabinose, d-galactose, d-glucose, d-glucuronic acid, and their 6-deoxy derivatives, such as fucose or rhamnose, as well as pentoses, such as arabinose, xylose, d-rhamnose, or a combination thereof [20,21].

### 3.2. Correspondence Between Antioxidant Capacity and Structure of Flavonoids

**Figure 4:**
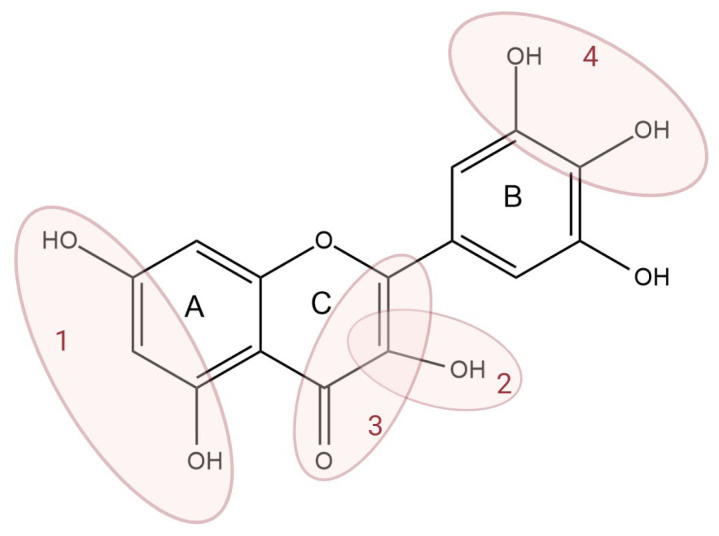
Structural relationship with the antioxidant activity of flavonoids. The increasing antioxidant effect resulted by: orto-hydroxyls on the A ring (at positions 5 and 7) (1); hydroxyl group at position 3 on the C ring (2); the double bond at the 2,3 position and the 4-keto group because of creating a conjugated system (3); two adjacent hydroxyl groups in B ring (4).

The antioxidant effect of flavonoids depends primarily on the presence of phenolic hydroxyl groups. The more free -OH groups there are on the molecule, the stronger the free radical scavenging ability in general. The ortho-dihydroxy (catechol) structure on the B ring further increases the radical scavenging ability (see Figure 4). A single hydroxyl on the B ring (for example, only in the 4’-position) provides moderate antioxidant activity, but two adjacent hydroxyl groups (3’,4’-dihydroxycatechol) significantly increase the effect [22,23].

The hydroxyl group at position 3 on the C ring of flavonoids also enhances the antioxidant effect. Flavonols (e.g., quercetin, kaempferol, and myricetin) also contain an -OH group at position 3 of the flavonoid C ring, in contrast to flavones (e.g., apigenin and luteolin), which lack this -OH [23]. Thus, the antioxidant activity of quercetin surpasses that of luteolin, which is a structural analogue of quercetin, only lacking the -OH at position 3 [22]. The same can be observed, for example, in the relationship between kaempferol (3,5,7,4’-tetrahydroxy flavonol) and apigenin (5,7,4’-trihydroxy flavone). Although both have a –OH group at position 4’ on the B ring, kaempferol is a stronger antioxidant due to the presence of the -OH at position 3; accordingly, in the measurements, the free radical scavenging activity of kaempferol is higher than that of apigenin [24].

The hydroxyls on the A ring (at positions 5 and 7) play a somewhat smaller role in free radical neutralization. The presence of two -OHs on the A ring (e.g., the 5,7-dihydroxy pattern in apigenin, luteolin, quercetin, etc.) allows for some degree of resonance stabilization, but these primarily increase the stability of the internal structure of the molecule, not directly the strongest radical-binding sites [23].

The saturation of the C ring of the flavonoid skeleton is also an important factor. The double bond at the 2,3 position and the 4-keto group create a conjugated system in the A–C rings, which allows the phenoxy radical of the B ring to delocalize throughout the molecule [23]. Flavonoids with such conjugation (flavones, flavonols) are typically stronger free radical scavengers than flavanones or flavanols with a saturated C ring. For example, naringenin (4’,5,7-trihydroxyflavanone)—which does not planarize due to the lack of a 2,3-bond—is a much weaker antioxidant than its structural analogue, apigenin (4’,5,7-trihydroxyflavone). Due to the lack of conjugation, the oxidation potential of naringenin is ~0.89 V, compared to ~0.82 V for apigenin or ~0.39 V for quercetin—the lower potential favors easier electron/hydrogen donation [25].

Methoxy (-OCH_3_) substituents are common among natural derivatives of flavonoids (e.g., 3’-methoxyquercetin = isorhamnetin, 7-methoxyquercetin = rhamnetin, diosmetin = 4’-methoxyluteolin, etc.). Based on IC_50_ values, quercetin achieves 50% DPPH (2,2-difenil-1-pikrilhidrazil) neutralization at a concentration of ~8.5 µg/mL, while rhamnetin requires ~53 µg/mL and isorhamnetin ~93 µg/mL for the same. Methoxylation reduces the number of free phenolic –OH groups and cannot donate hydrogen to the free radical, thus reducing the number of possible hydrogen donor sites and thus the antioxidant capacity [22].

The situation is similar for glycosides. Flavonoids are often found in plants bound to a sugar molecule (in the form of *O*- or *C*-glycosides). The glycosidic bond—especially the *O*-glycoside, which typically binds a phenolic -OH—also reduces the antioxidant effect. The sugar linkage occupies an -OH group, so it cannot participate in free radical neutralization [26]. A comparison of quercetin and rutin (quercetin-3-*O*-rutinoside) illustrates this well: the quercetin aglycon has more “reactive centres” and is therefore much more active in redox reactions, while in the case of rutin, the antioxidant activity is weaker due to the ether bond of the -OH at position 3 to the sugar [27].

### 3.3. Influence of Complexation with Cyclodextrins on the Antioxidant Activity of Flavonoids

The typical framework of flavonoids is a 2-phenylchromenone-type aromatic system, which is planar and hydrophobic, so its size and shape fit well into the hydrophobic cavity of the cyclodextrin. The aromatic rings of the guest flavonoid are wedged into the cavity of the host cyclodextrin through hydrophobic interactions, displacing water molecules [28].

Studies have shown that the B-ring of the flavonoid molecule is often oriented towards the wider (secondary) opening of the β-cyclodextrin in the complex, highlighting the important structures responsible for the antioxidant effect of the flavonoid guest molecule [29]. Flavonoids contain multiple hydroxyl groups that can form hydrogen bonds with hydroxyl groups at the opening of the cyclodextrin cavity, further stabilizing the complex. Due to these structural features, flavonoid–cyclodextrin complexes typically appear as stable, 1:1 stoichiometric inclusion complexes that are significantly more soluble in water than free flavonoids [28].

**Figure 5:**
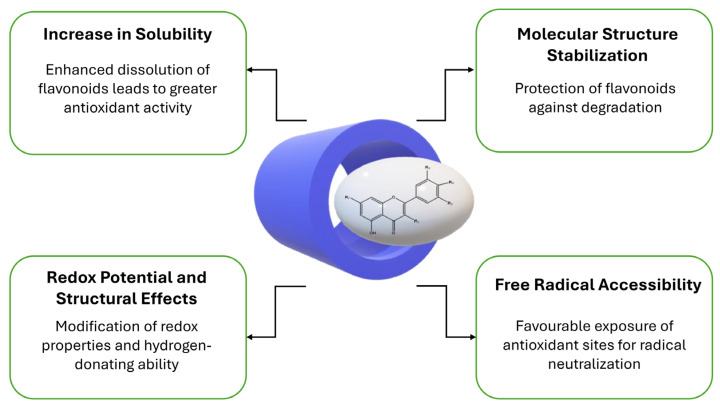
The consequence of guest–host complexation is due to the antioxidant effect of flavonoids.

Cyclodextrins can accommodate hydrophobic compounds as hollow, barrel-shaped molecules. This host–guest complexation offers a new opportunity to improve the solubility and stability of flavonoids without modifying their chemical structure [30]. This review presents, based on the literature, how complexation with cyclodextrins affects the antioxidant capacity of flavonoids, what the main mechanisms behind this are, and in which cases the antioxidant effect of flavonoids is enhanced or weakened by complexation (see Figure 5). Specific examples are discussed for different flavonoid groups and CD types.

#### 3.3.1. Increase in Solubility

Many flavonoids have low water solubility, which limits their antioxidant activity in aqueous media. The main advantage of cyclodextrin complexes is that they can significantly increase the solubility of the guest molecule in the aqueous phase. For example, the methylated β-CD complex of quercetin (with a >254-fold increase in solubility) allowed the presence of much higher concentrations of quercetin in aqueous media, which increased its DPPH radical scavenging activity by about 10% compared to free quercetin [31]. Similarly, the formation of the β-CD complex of rutin reduced the EC_50_ value (the concentration required to bind 50% of the DPPH radical) from 1.547 × 10^−5^ M to 1.227 × 10^−5^ M, indicating an increase in antioxidant activity [30]. The improvement in solubility, therefore, makes more active flavonoid molecules available for the reaction, thereby increasing the measured antioxidant capacity. Overall, if the solubility of the flavonoid is the limiting factor, cyclodextrin complexation typically enhances the antioxidant capacity by allowing more molecules to react with free radicals.

#### 3.3.2. Molecular Structure Stabilization

The cavity of cyclodextrins not only increases solubility but can also serve as a protective shell for the guest flavonoid. Complexation can stabilize the flavonoid molecule against heat, light, or oxygen, preventing its degradation or inactivation [32]. As a result, the antioxidant effect can be maintained for a longer period and is less degraded at higher temperatures or during storage. In one study, complexing antioxidant flavonoids from citrus fruits with HP-β-CD increased their thermal stability from 120 °C to ~250 °C [32]. In addition, the incorporation of the complexes into a biopolymer film also improved the temporal stability of the antioxidant activity: CD-complexed antioxidants retained their radical scavenging ability three times longer than the uncomplexed control. Stabilization, therefore, indirectly contributes to the maintenance of antioxidant capacity, especially during long-term studies or storage. However, it is important to note that the stabilizing effect can sometimes slow down the reaction rate: the flavonoid becomes more protected and can react with radicals more slowly. In such cases, the immediately measured antioxidant activity may be temporarily lower, but in the long term, more active molecules remain in the system.

#### 3.3.3. Redox Potential and Structural Effects

Complexation can subtly modify the chemical environment of the flavonoid, which can affect its redox potential and hydrogen-donating capacity. In some cases, cyclodextrin–flavonoid interactions can facilitate the oxidation (electron or hydrogen donation) of the flavonoid, resulting in a stronger antioxidant effect. In the case of quercetin, it has been shown that in the cyclodextrin complex, new intermolecular hydrogen bonds are formed between the hydroxy groups of CD and quercetin, which break the intramolecular H-bonds within the quercetin molecule [33]. Due to the weakening of intramolecular H-bonds, quercetin- and analogously other flavonoids-donate hydrogen to free radicals more easily, thereby increasing their antioxidant efficiency. This was also supported by the study of Liu et al., who reported that the increase in antioxidant activity of quercetin in complexes with three different β-CD derivatives can be explained by the increased hydrogen-donating capacity. The change in redox potential is often followed by electroanalytical methods (e.g., cyclic voltammetry), although specific potential values are rarely reported in the literature. However, the tendency is that a properly formed complex slightly reduces the first oxidation potential of the flavonoid (it is more easily oxidized), which is consistent with a stronger radical scavenging ability. It should be noted that this effect is not always positive: if certain reactive groups of the flavonoid are deep in the CD cavity, they may contribute less to electron release, i.e., the redox potential is not improved in a practical sense [30].

#### 3.3.4. Free Radical Accessibility

In flavonoid–CD complexes, the flavonoid molecule is partially or completely located in the cyclodextrin cavity. This affects how easily free radicals (or indicator radicals used in the measurement, e.g., DPPH^●^ or ABTS^●+^) can access the flavonoid radical scavenging sites. In the preferred case, the flavonoid sits in the cyclodextrin in the correct orientation, so that the key hydroxyl radicals protrude from the cavity and easily react with the radicals. For example, in the case of luteolin (LU) and β-CD derivatives, molecular modelling has shown that the B-ring of the flavonoid (which carries the 3’,4’-dihydroxy groups) in certain complexes (e.g., HP-β-CD) is oriented towards the wide opening, i.e., more accessible [34]. In line with this, ESR (spin trapping) radical scavenging experiments show that the antioxidant activity of luteolin complexes is LU–HP-β-CD > LU–DM-β-CD > LU–β-CD > free luteolin, indicating that the better the accessibility of the flavonoid in the complex, the more efficient the radical neutralization. In an unfavourable case, however, the flavonoid may become too immersed in the cyclodextrin, and radicals (especially larger DPPH˙ molecules) do not fit into the cavity or reach the reactive sites more slowly [30]. The accessibility of free radicals is also influenced by the antioxidant measurement methods used: more polar radicals (such as the ABTS^●+^ ion) diffuse more easily into the vicinity of the complex in the aqueous phase, while the behaviour of the hydrophobic DPPH˙ radical may be different in organic vs. aqueous media.

### 3.4. Prooxidant Effect of Flavonoids and Their CD Complexes

**Figure 6:**
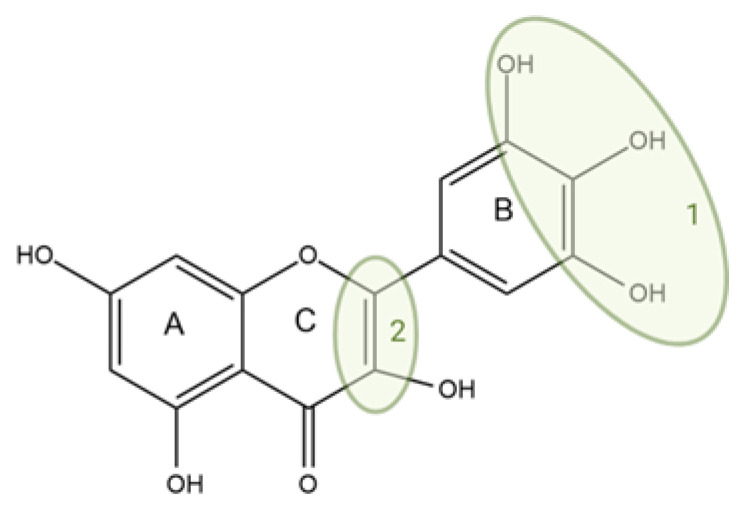
Relationship of chemical structure and prooxidant activity. Dihydroxyl-trihydroxyl B ring (1) and double bond in the C ring (2) increase the prooxidant effect.

Flavonoids are primarily known for their strong antioxidant effects, which refers to their ability to scavenge free radicals within cells, thereby reducing oxidative stress and inflammation in the body, but in some cases, they can also exert an enhanced prooxidant effect. Through their prooxidant properties, polyphenol compounds can cause oxidative damage by reacting with various biomolecules such as proteins, lipids, and DNA. This dual effect depends on their structure, concentration, and interaction of the flavonoids with other antioxidants in the body [1]. The prooxidant activity of flavonoids is thought to be directly proportional to the total number of hydroxyl groups in the flavonoid molecule (Figure 6) [35]. A series of mono- and dihydroxy flavonoids were shown to have no detectable prooxidant activity. At the same time, molecules with multiple hydroxyl groups, especially in the B-ring, significantly increased the amount of hydroxyl radicals in the Fenton reaction [36]. The prooxidant properties of flavonoids depend on their concentration, as well. For example, Yen et al. investigated the prooxidant properties of quercetin, naringenin, hesperetin, and morin in human lymphocytes. Naringenin and hesperetin promoted Fe(^3+^)/H_2_O_2_-induced deoxyribose oxidation at concentrations ranging from 0 to 200 µM, while quercetin and morin suppressed it at concentrations above 100 µM [37]. Prooxidant activity is exhibited by easily oxidizable, low-molecular-weight polyphenols, such as quercetin and gallic acid. In contrast, high-molecular-weight phenols, such as hydrolysable and condensed tannins, have little or no prooxidant properties [38].

Flavonoids are increasingly being investigated as anticancer agents due to their prooxidant effects. In the process of carcinogenesis, flavonoids can influence numerous signalling pathways, suppress proliferation, angiogenesis, and metastasis, or enhance apoptosis. For example, luteolin is effective in therapeutically induced cytotoxicity of cancer cells due to its suppression of cell survival pathways, such as phosphatidylinositol-3′-kinase (PI3K)/Akt, NF-κB, and X-linked inhibitor of apoptosis protein (XIAP), resulting in the stimulation of apoptosis pathways, including the induction of the p53 tumour suppressor [39].

The influence of cyclodextrins on the prooxidant or antioxidant effects of flavonoids depends on the specific flavonoid, the type of cyclodextrin, and the environment. It was reported that cyclodextrin–flavonoid complexes can enhance mitochondrial permeability transition, a process linked to oxidative stress, suggesting a possible increase in prooxidant activity under certain conditions [40]. Most studies show that cyclodextrin inclusion generally increases the antioxidant activity of flavonoids by improving their solubility and stability in aqueous environments. The effect of cyclodextrin inclusion can vary with solvent polarity. In polar environments, the antioxidant effect is enhanced. For example, apigenin, dihydromyricetin, quercetin, and rutin all showed increased radical scavenging activity when complexed with various cyclodextrins, especially in polar (water-based) environments [41,42,43,44]. However, in less polar environments, cyclodextrin can reduce the antioxidant activity of flavonoids by stabilizing their structure and making hydrogen donation less favourable, which could theoretically shift the balance toward a prooxidant effect [9]. When multiple flavonoids are present, cyclodextrin complexation can alter the ratio of individual flavonoids, sometimes resulting in lower overall antioxidant and thus potentially higher prooxidant activity compared to the uncomplexed mixture [45]. The multicomplexation of quercetin, luteolin, and 3-*O*-methylquercetin was investigated, simultaneously present in the flavonoid-enriched fraction of *Achyrocline satureioides* with HP-β-CD. Using the phase solubility diagram, a linear correlation was demonstrated between the solubility of flavonoids and the concentration of HP-β-CD, indicating the formation of complexes with a 1:1 stoichiometric ratio, which was determined by Electrospray Ionization Mass Spectrometry (ESI-MS) [45].

## 4. Flavonoid–Cyclodextrin Complexes

### 4.1. Flavanon–Cyclodextrin Complexes

Flavanones are primarily synthesized in citrus fruits and are most commonly found in glycosidic form. The main aglycones include naringenin (the 7-*O*-glycoside is naringin) found in grapefruit, hesperetin (the 7-*O*-glycoside is hesperidin) found in oranges, and eriodyctiol found in lemons. Flavanones (also known as dihydroflavones) have a saturated C-ring. The saturation between positions 2 and 3 of the C-ring represents the structural difference compared to other flavonoid groups [46].

Flavanones are poorly soluble in water, unstable compounds, which limits their biological utilization [47]. During complexation with cyclodextrins (CD), α-, β-, and γ-cyclodextrins can accommodate the apolar parts of flavanone molecules into their hydrophobic inner cavities. The “inclusion” of guest molecules is achieved through weak non-covalent interactions (hydrophobic and van der Waals interactions, H-bonds) [48]. As a result of cyclodextrin complexation, the water solubility and chemical stability of flavanones are improved, which overall increases their biological activity [47]. The most common stoichiometry of cyclodextrin–flavanone complexes is the 1:1 ratio, which is also indicated by linear A_L_ curves of phase solubility (Higuchi–Connors) studies [48]. Flavanon-CD complex stability and antioxidant capacity are shown in Table 1.

However, in some cases, higher-order complexes can also be formed: for example, complexes with a ratio of 1:2 (one flavanone molecule partially inserted into two CD cavities) or 2:1 (two guest molecules in a larger host cavity) have been described under appropriate conditions [49]. The efficiency of complex formation is greatly influenced by the type of cyclodextrin. According to experimental data, the stability constants (K) of flavanones are highest with β-CD derivatives. For example, the complexation ability of HP-β-CD with different degrees of substitution exceeded that of native β-CD (and surpassed HP-α- and HP-γ-CD). Modified β-CDs—such as methylated derivatives (RAMEB, DM-β-CD)—are particularly effective in complexing flavanones due to their increased water solubility and more favourable conformation [50]. However, it is possible that steric hindrances may moderately reduce the binding strength of some substituted CDs compared to native β-CD [48].

The stability of the complexes is mainly determined by the size complementarity of the host–guest and the hydrophobic nature of the flavanone molecule: stronger hydrophobic guests give higher K values [50]. Typical stability constants are of the order of 10^3^ M^−1^; for example, in the case of the hesperetin–β-CD complex, K is 2−3 × 10^2^ M^−1^, while with RAMEB it is ~1–1.3 × 10^3^ M^−1^. The formation of stable complexes significantly increases the apparent water solubility of flavanones: in the case of naringenin, the solubility of the free form, ~4 μg/mL, increased to ~1.5 mg/mL upon complexation in the presence of methylated β-CD derivatives, i.e., a solubility enhancement of several hundredfold can be achieved [47].

**Table 1:** Stability and antioxidant activity of flavonoid–cyclodextrin complexes.

	CD-Derivate	Complex Ratio	Complexation Constant (K, M^−1^)	Antioxidant Activity	Reference
**Flavanons**					
**Naringenin**	β-CD	1:1	450	↑ ^1^	[51]
	HP-β-CD	1:1	4000	const. ^2^	[52]
	Me-β-CD	1:1/1:2	2100	const.	[49]
	γ-CD	1:1	600	↓ ^3^	[53]
**Hesperetin**	β-CD	1:1	300	↓	[51]
	Me-β-CD	1:1	1800	↑	[54]
**Flavons**					
**Apigenin**	β-CD	1:1	1830	↑↑ ^4^	[55]
	γ-CD	1:1		↑↑	[56]
	HP-β-CD	1:1	4510	↑↑	[55]
	Me-β-CD	1:1	≈1040	↑	[55]
**Luteolin**	β-CD	1:1	≈2330	↑	[55]
	HP-β-D	1:1	≈51,400	↑↑	[55]
	Me-β-CD	1:1	≈4460	↑	[55]
**Chrysin**	β-CD	1:1	275	↑	[57]
	HP-β-CD	1:1	760	↑↑	[57]
	SBE-β-CD	1:1	1000	↑↑	[57]
	RAMe-β-CD	1:1	1200	↑↑	[57]
**Flavonol**					
**Kaempferol**	β-CD	1:1	580	↑	[58]
	HP-β-CD	1:1	6200	↑	[58]
	Me-β-CD	1:1	5400	↑	[58]
**Quercetin**	β-CD	1:1	≈600	↑	[59]
	HP-β-CD	1:1	≈1400	↑	[59]
	SBE-β-CD	1:1	≈4000	↑↑	[59]
**Myricetin**	β-CD	1:1	617	N/A ^5^	[60]
	HP-β-CD	1:1	3090	N/A	[60]
	Me-β-CD	1:1	1250	N/A	[60]
	γ-CD	1:1	390	N/A	[60]
**Rutin (kvercetin-3-*O*-rutinozid)**	β-CD	1:1	≈250	↑	[61,62]
	HP-β-CD	1:1	≈390	↑	[61,62]
**Fizetin**	β-CD	1:1	≈900	N/A	[63]
**Morin**	β-CD	1:1	396	↑	[64]
	HP-β-CD	1:1	1408	↑	[64]
	Me-β-CD	1:1	1700	↑	[64]
**Anthocyanins**					
**Pelargonidin**	α-CD	1:1	unstab.	N/A	[65]
	β-CD	1:1	N/A	N/A	[65]
	γ-CD	1:1	↑	N/A	[65]
	Me-β-CD	1:1	stabile	N/A	[65]
**Cianidin-3-*O*-glükozid**	α-CD	-	unstab.	-	[66]
	β-CD	1:1	↑	-	[66]
**Malvidin-3-*O*-(6-*O*-p-kumaroil-glükozid)**	γ-CD	1:1	↑↑	const.	[66]
**Delphinidin**	SBE-β-CD	1:1	↑↑	const./ ↑	[67]
**Isoflavonoids**					
**Genistein**	β-CD	1:1	≈3.5 × 10^3^	const./ ↑	[68]
	HP-β-CD	1:1	≈1.1 × 10^4^	↑	[69]
	SBE-β-CD	1:1	≈3.5 × 10^4^	↑	[69]
**Daidzein**	β-CD	1:1	≈7.8 × 10^2^	↑	[70]
	HP-β-CD	1:1	≈1.8 × 10^3^	↑↑	[70]
**Chalcones**					
**4’-hydroxy-chalcone**	β-CD	1:1	≈4.8×10^2^	N/A	[71]
	HP-β-CD	1:1	≈9.9×10^2^	N/A	[71]

^1^ Weak–moderate increase in antioxidant capacity due to complexation based on the reference. ^2^ Antioxidant capacity is unchanged by complexation. ^3^ Weak–moderate decrease in antioxidant capacity due to complexation based on the reference. ^4^ Moderate–strong increase in antioxidant capacity due to complexation based on the reference. ^5^ Data is not available.

### 4.2. Flavon–Cyclodextrin Complexes

Flavones (e.g., apigenin, luteolin, and chrysin) are polyphenolic compounds that are poorly soluble in water; therefore, their oral bioavailability is limited [56]. Complexation with cyclodextrins is an effective method to alleviate this problem, in which the stoichiometry is mostly 1:1 [57]. The driving force of complexation is the hydrophobic effect: the aromatic part of the flavone (e.g., the B-ring of luteolin) fits into the apolar cavity of the CD, displacing water molecules. In addition, van der Waals forces and hydrogen bonds can also stabilize the complex [33].

Several examples of flavone–CD complexes are known (see Table 1). In the case of apigenin, complexes with β-CD and its derivatives (e.g., HP-β-CD, SBE-β-CD, and Me-β-CD) increase the solubility and antioxidant activity of apigenin by orders of magnitude; typically, 1:1 complexes are formed (e.g., HP-β-CD), but in the case of the higher affinity Me-β-CD, even 1:2 stoichiometry can occur [56]. Similarly, luteolin forms 1:1 complexes with different β-CD forms, and the stability constant was highest for glycosylated β-CD [26].

Luteolin is a 3’,4’-dihydroxyflavone with significant antioxidant potential, but similar solubility limitations to quercetin. β-CD, HP-β-CD, and dimethyl-β-CD (DiMe-β-CD) complexes were prepared from luteolin and characterized by fluorescence and NMR spectroscopy [34]. The stability order was HP-β-CD > DiMe-β-CD > β-CD, which also corresponded to the degree of solubility improvement. Antioxidant activity was assessed by ESR (electron spin resonance) spectroscopy, probably by monitoring the disappearance of DPPH or similar radicals. According to the results obtained, all three luteolin complexes were more potent antioxidants than free luteolin, in the following order: luteolin–HP-β-CD > luteolin–DiMe-β-CD > luteolin–β-CD > luteolin (free). For example, HP-β-CD complexed luteolin almost completely neutralized the tested radicals in the experimental concentration range, while free luteolin only partially [34].

The better effect was partly explained by the fact that the B-ring of luteolin in the HP-β-CD complex is located at the wider opening of the CD, so that the 3’,4’-dihydroxy groups can easily react with radicals. Furthermore, in the case of HP-β-CD, the more stable complex also protects luteolin better from oxidative degradation, so its effect is more durable. In the case of luteolin, therefore, complex formation clearly enhances the antioxidant effect, especially when using a properly chosen derivative (HP-β-CD). It is worth noting that the antioxidant effect of luteolin itself is considerable—according to some studies, it may be stronger than that of vitamin C or E—so cyclodextrin rather promotes the full utilization of this potential in an aqueous medium [34].

The research examined various β-CD derivatives, including HP-β-CD, DM-β-CD, and HP-β-CD-1, to identify the most effective derivative for forming stable inclusion complexes with luteolin. The study employs molecular docking, molecular dynamics (MD) simulations, MM-PBSA, umbrella sampling, and quantum mechanics calculations to analyze the binding affinity, structural stability, and thermodynamics of luteolin-CD inclusion complexes. It was revealed that derivatives like HP-β-CD-1, which contain more hydroxypropyl groups, provide a larger and more optimized binding cavity. This leads to stronger binding affinity and better encapsulation of luteolin, further improving its solubility. This study demonstrated that HP-β-CD-1 is the most effective β-CD derivative for improving luteolin solubility [72].

The chrysin solubilization capacity of different β-cylcodextrin derivatives was investigated and compared to their biological activities. Chrysin was complexed with β-cyclodextrin (β-CD), hydroxypropyl-β- (HP-β-CD), sulfobutylether-β-CD (SBE-β-CD), and randomly methylated-β-cyclodextrin (RAMEB) by the lyophilization method in 1:1 and 1:2 molar ratios. The solubility increment was the highest by RAMEB (more than seven times), and the complex stability and solubility increased by CD derivatives in the following order (for 1:1 complexes): β-CD < HP-β-CD < SBE-β-CD < RAMEB. In the permeability study the RAMEB 1:1 and 1:2 complexes were the most effective to enhance chrysin permeability through the Caco-2 monolayers. In conclusion, RAMEB was the most suitable for increasing the bioavailability of chrysin [57].

### 4.3. Flavonol–Cyclodextrin Complexes

Flavonols (e.g., quercetin, kaempferol, and rutin) typically form inclusion complexes with cyclodextrins. In these, the flavonol molecule fits into the cavity of the cyclodextrin, with its hydrophobic part in the inner cavity, while the polar groups are oriented outward. The most common stoichiometry is the 1:1 complex, i.e., one flavonol molecule forms a stable host–guest relationship with one cyclodextrin molecule (see in Table 1). For example, the solubility profile of kaempferol with various cyclodextrins (β-CD and its derivatives) is linear (Higuchi–Connors A_L_ type), which indicates a 1:1 molecular ratio complex formation [60]. However, in some cases, 1:2 complexes can also form: in the case of larger or less fitting flavonols, two cyclodextrins can bind to one guest. Such a phenomenon was observed in the case of rutin and β-CD, where FT-IR, DSC, and X-ray diffraction studies also revealed the presence of rutin–β-CD complexes with 1:1 and 1:2 molar ratios [73].

The stability of cyclodextrin complexes is characterized by stability (association) constants, which indicate the strength of the binding between the flavonol and the cyclodextrin. These constants usually fall in the range of 10^2^ – 10^4^ M^−1^, indicating a moderately strong but reversible interaction. The type of cyclodextrin strongly influences the stability: in general, modified β-CD derivatives (e.g., HP-β-CD, Me-β-CD, and SBE-β-CD) form stronger complexes than native β-CD [74]. For example, in the case of kaempferol, the stability constant with HP-β-CD was ~2312 L/mol, while with native β-CD it was ~551 L/mol [60]. Similarly, very high K values of the order of 10^4^ were also measured for the complex of quercetin and SBE-β-CD [75]. The stability of the complexes also depends on the pH: flavonols often bind more stably in acidic media. For example, the stability constant of kaempferol-4’-O-glucoside at pH 6.0 is higher than that at pH 7.4 or 9.0 with the same cyclodextrin, suggesting that the ionization state of the flavonol influences the binding [74]. One of the main advantages of complex formation is that it increases the chemical stability of flavonols. The cyclodextrin “shell” protects the guest molecule from environmental effects: its thermal and photostability increase. According to experiments, the quercetin–HP-β-CD complex showed a slight improvement in the photostability of quercetin compared to free quercetin, i.e., it degraded more slowly under the influence of UV light [76]. Similarly, a decrease in degradation in the presence of heat or light has been observed for other flavonol complexes, as the cyclodextrin partially protects the molecule from harmful effects. In addition, complexation can dramatically increase the water solubility of flavonols—for example, the solubility of quercetin can increase several hundredfold in the presence of HP-β-CD—which also indirectly has a stabilizing effect (reducing loss due to precipitation or decomposition) [77].

The reviewed literature has consistently found that the formation of inclusion complexes is an effective strategy for increasing the stability of flavonols. The most important trends include the dominance of 1:1 complex, the beneficial effect of modified β-cyclodextrins on the binding strength, and the improvement of stability parameters (higher stability constants, increased photo- and thermal stability, favourable pH tolerance, etc.). These complexes are not only more stable, but often also more bioavailable, since the increased solubility and stability give the flavonols a better chance of retaining their activity and reaching their target during use [74,76,77].

### 4.4. Anthocyanin–Cyclodextrin Complexes

Anthocyanins are mostly water-soluble plant pigments from the flavonoid group, which have significant antioxidant and other beneficial biological effects. However, they are chemically unstable compounds: they are sensitive to pH changes, light, and temperature, which causes their degradation and loss of colour [78]. This instability, as well as their sometimes poor solubility (aglycone form) and low bioavailability, limit their use in the food industry or medicine [67,78].

The internal cavity of cyclodextrins is hydrophobic, thus able to accommodate the aromatic rings of anthocyanins. Anthocyanin–CD complexes are typically a 1:1 stoichiometric inclusion complex, in which the anthocyanin aglycone partially fits into the CD cavity. For example, the neutral hemiketal form of cyanidin-3-O-glucoside (C3G) forms a 1:1 complex with β-CD, where the pyran C ring of the anthocyanin fits deeply into the β-CD cavity, while the B ring is located at the wide edge of the β-CD [79]. The mechanism of complex formation is mainly based on hydrophobic interactions and hydrogen bonds. Computer modelling has shown that anthocyanins containing two sugar units (rutinosides) bind more strongly to CDs than monoglycosides, since the more hydroxyl groups form a more stable hydrogen bond network. The type of CD also influences the interaction: β- and γ-cyclodextrin are more favourable for anthocyanins, while α-CD is less effective due to its smaller cavity [80].

The pH-dependent equilibrium forms of anthocyanins—e.g., the red flavylium cation, the bluish quinoid base, and the colourless hemiketal and cis/trans-chalcone forms—complex differently. According to NMR studies, β-CD does not interact with the flavylium cation of the C3G anthocyanin, but selectively accepts the hemiketal and cis-chalcone forms [79]. Accordingly, the association equilibrium constant for the flavylium forms is negligible, while the neutral base and open-chain chalcone forms already have a significant (≈10^2^–10^3^ M^−1^) binding constant [79,81]. Complexation, therefore, also affects the colour and structural equilibria: the presence of β-CD can shift the internal equilibrium of the anthocyanin towards the colourless forms by binding the chalcone forms (co-pigmentation phenomenon), thereby reducing the immediate colour intensity but increasing the long-term stability [79]. Empirical measurements have shown that the addition of cyclodextrin significantly improves the heat and light stability of anthocyanins [78]. For example, in the case of mulberry anthocyanins, the use of β-CD increased the activation energy of thermal decomposition by ~20% (from 63.06 kJ/mol to 76.77 kJ/mol), while in the case of black bean hull anthocyanins, the presence of β-CD in the beverage extended the half-life of the anthocyanins extremely, up to 13 months [73,78]. The colour stability measured during storage of the same complexes improved: the colour intensity and hue changed only minimally compared to the control [73]. Based on all this, cyclodextrin complexation exerts a protective effect on the anthocyanins, slowing down their oxidative and thermal degradation.

In the case of anthocyanins, the investigation of complexes formed with individual substances is less typical; they form complexes with anthocyanin extracts of various plant and fruit extracts. Therefore, the stability constant of anthocyanin complexes is not included in Table 1; only the characterization given in the literature is included.

The strong free radical scavenging (antioxidant) activity of anthocyanins is preserved by cyclodextrin complexes and even further utilized by increasing stability. Cyclodextrin-protected anthocyanins can exert their antioxidant effect for a longer period, for example, in foods or dietary supplements, without rapidly degrading. In the case of β-CD complexes of anthocyanins extracted from black bean skins, it was shown that the extracts produced in this way have high antioxidant potential, while they can also be used as natural colorants [73]. Similarly, cyclodextrin-assisted extraction increased the amount of anthocyanin recovered and its antioxidant activity from mulberry fruits [78]. An important pharmacological advantage is that complex formation improves the water solubility and bioavailability of anthocyanins. In the case of a delphinidin–SBE-β-cyclodextrin (sulfobutyl ether-β-CD) complex, the anthocyanin administered in a stable, water-soluble form was absorbed much more efficiently and could exert a biological effect than free delphinidin [67]. The complex was tested in an experimental inflammatory pain model and was found to have significant anti-inflammatory and analgesic effects, due to the antioxidant activity of anthocyanin. Interestingly, cyclodextrin not only acts as a carrier in this case but also provides a dual protective mechanism: the cavity of the CD is able to bind certain harmful oxidation by-products (e.g., the lipid peroxidation product called 4-hydroxy-2-nonenal), and then releases the guest anthocyanin, which neutralizes the resulting free radicals. Thus, the cyclodextrin–anthocyanin complex simultaneously protects against oxidative damage and ensures the controlled presence of the anthocyanin active ingredient at the target site [67]. Overall, complexation increases the effectiveness of anthocyanins: they can be used in a more stable form, with unchanged antioxidant capacity, which is especially valuable in the development of functional foods and pharmacological preparations.

### 4.5. Isoflavonoid–Cyclodextrin Complexes

Isoflavonoids and cyclodextrins generally form inclusion complexes with a 1:1 stoichiometry. This is supported by phase solubility studies, according to which the diagram type is A_L_ (linear isotherm, slope < 1) and the Job method—for example, in the case of daidzein, the phase diagram is straight, with a slope < 1 and a maximum at a molar ratio of 0.5, indicating 1:1 complex formation. The stability of the complex is characterized by the complex formation (association) constant (K), which typically falls in the range of 10^2^–10^4^ M^−1^ for these guest molecules, depending on the type of CD and the structure of the guest (see Table 1). Native β-CD generally shows a relatively lower binding affinity compared to hydrophilic modified derivatives. For example, the K_app_ value of the daidzein–β-CD complex is ca. 776 M^−1^, while the complexes of the same isoflavone with methylated β-CD (Me-β-CD) and 2-hydroxypropyl-β-CD (HP-β-CD) are much more stable (K ≈ 1418 and 1802 M^−1^, respectively). This is because the methylation or hydroxypropylation of β-CD increases the effective opening of the CD cavity and weakens the internal H-bond network of CD, so that the guest molecule can be embedded more easily, forming a stronger complex [70,82].

A similar trend can be observed in the case of genistein: while literature data for the genistein–β-CD complex give a K ≈ 3.5 × 10^3^ M^−1^, with randomly methylated β-CD (RAMEB) or HP-β-CD, the stability is on the order of ~1 × 10^4^ M^−1^. Moreover, even higher binding is achieved with certain modified β-CDs—e.g., in the case of sulfobutyl ether-β-CD (SBE-β-CD), the complexation constant of genistein can approach 3.5 × 10^4^ M^−1^, compared to ~1.3 × 10^4^ M^−1^ for daidzein for the same CD. These data indicate that genistein, due to its larger size and more hydrophobic nature, may bind more strongly to certain cyclodextrins than daidzein [68,69,82].

NMR and molecular modelling studies provide insight into how isoflavonoid molecules are positioned in the CD cavity. In the case of daidzein, ^1^H–^1^H ROESY experiments show that primarily the B ring (the aromatic ring of the isoflavone, which contains the 4’-hydroxyl group) is immersed in the β-CD cavity. In contrast, in the complexes of genistein, which also has an extra 5-hydroxyl group, multiple orientations are possible. Some studies suggest that genistein tends to insert its A ring (the other aromatic ring of the isoflavone) into the β-CD cavity, although other results do not exclude entry via the B ring. 2D-NOESY NMR experiments have shown that the protons of the A ring of genistein come into spatial proximity with the protons of the internal H3/H5 of β-CD, supporting the entry of the A ring into the cavity [68,70].

One of the most important practical advantages of cyclodextrin complexes is the intense increase in solubility. The water solubility of hydrophobic isoflavonoids can be increased by orders of magnitude by complexation. For example, in the case of daidzein, the water solubility increases almost tenfold in the presence of the complexing agent HP-β-CD: a ~9.7-fold increase in solubility was measured when 5 mM HP-β-CD was added, while the same concentration of β-CD resulted in only a ~4.8-fold increase (with Me-β-CD being an intermediate ~8.1-fold) [70].

In the case of a sulfobutyl ether-β-CD (SBE-β-CD, sodium salt), the solubility of genistein and daidzein is also orders of magnitude better than that of native β-CD, paralleling higher stability constants. According to the experiments, the antioxidant effect of isoflavonoid–CD complexes is typically not reduced, and in fact often increases, compared to the free isoflavonoid. As a specific example, daidzein complexes were examined using the DPPH free radical scavenging test. The measurements showed that the daidzein cyclodextrin complexes have a significantly stronger free radical scavenging effect than native daidzein, the order of which is: Daidzein–HP-β-CD > Daidzein–Me-β-CD > Daidzein–β-CD > free daidzein, i.e., the HP-β-CD complex showed the strongest effect [70]. While native daidzein was only able to eliminate DPPH radicals to a limited extent at a given concentration, the complexed form became much more effective. One explanation for this is that complex formation increases the H-donor ability of the isoflavonoid. For example, HP-β-CD strongly interacts with the daidzein molecule, breaking the intramolecular H-bonds within the daidzein molecule and forming guest–host H-bonds instead. This “internal stress release” makes the phenolic hydroxyl groups more reactive, so they more easily donate protons to the free radical. Interestingly, it was observed that the effect was not as prominent in the case of CDs containing too many hydrophobic substituents (e.g., highly methylated β-CD) because the more methyl groups weakened the H-bonds between the guest and CD, which somewhat impaired the improvement in hydrogen-donating ability. This indicates that the stability of the complex and the mode of binding are crucial for enhancing the antioxidant effect: the strongest complex (daidzein–HP-β-CD) gave the highest DPPH neutralization effect, consistent with the binding order [70,82].

### 4.6. Chalcone–Cyclodextrin Complexes

Chalcones are aromatic compounds belonging to the family of α, β-unsaturated ketones, which contain two aromatic rings connected by a triple carbon chain. Several natural chalcones (e.g., isoliquiritigenin, cardamonin, etc.) are known for their biological effects, including antioxidant, anti-inflammatory, and antitumor activities. However, chalcones are generally poorly soluble in water or practically insoluble, which significantly limits their biological utilization and application [21,28,83].

Typically, complexes with a 1:1 stoichiometry are formed with cyclodextrins, as indicated by the A_L_-type curve of phase solubility diagrams. For example, 4′-hydroxychalcone and β-CD have been observed to form a 1:1 complex, similar to most chalcones (see Table 1) [28].

In general, the appropriately sized cavity of β-CD and its derivatives fits well with aromatic guest molecules, while α-CD (smaller cavity) has a weaker binding, and γ-CD (larger cavity) often has a weaker binding. In the case of HP-β-CD, even stronger complexes are often formed than with native β-CD. Guo and co-workers measured a stability constant of K ≈ 4.8 × 10^2^ M^−1^ for β-CD at 20 °C, while K ≈ 9.9 × 10^2^ M^−1^ for HP-β-CD, meaning that the substituted cyclodextrin bound the chalcone almost twice as strongly. Among the secondary forces stabilizing the complexes, van der Waals and hydrophobic interactions dominate, but in some cases, hydrogen bonds can also form between the cyclodextrin and the chalcone, especially if the polar (e.g., -OH) substituents of the chalcone are appropriately positioned [71].

Phase solubility studies can be used to determine the stoichiometry of the resulting complex and the degree of solubilization. Typically, linear (A_L_ type) curves are obtained, indicating a 1:1 complex formation ratio, but for certain guests with larger or two distant aromatic rings, a 1:2 ratio (one guest–two CDs) can also occur [84]. The increase in solubility for chalcones can be several orders of magnitude. For example, the water solubility of isoliquiritigenin (a natural 2′,4′,4-trihydroxychalcone) is only ~13.6 µM (on the order of 3 × 10^−6^ M) in pure form, which is considered extremely low. However, when complexed with SBE-β-CD, the solubility reaches a concentration of 4.05 mM [85]. This represents a nearly 300-fold increase in solubility due to the cyclodextrin complex.

It is worth noting that complexation can have a beneficial effect not only on the stability of the complex, but also on the stability of the guest molecule: the chalcone often becomes more protected from environmental influences. The thermal stability of 4′-hydroxychalcone was dramatically increased by complexation: in its pure form, it decomposed at ~200 °C, while in the HP-β-CD complex, the onset of decomposition was ~397 °C [71].

The antioxidant capacity of chalcones, which are barely soluble in water in the free form, is limited by their poor accessibility in aqueous media; in contrast, in the form encapsulated by cyclodextrin and dispersed in water, the chalcone molecules can react with free radicals to a greater extent. A specific example is again isoliquiritigenin: the in vitro antioxidant activity of isoliquiritigenin complexed to SBE-β-CD was many times that of its free form—the IC_50_ value of the DPPH free radical scavenging activity of the complexed form decreased to 42.2 μg/mL, indicating significantly better efficacy [85].

## 5. Formulation Options of Flavonoid-CD Inclusion Complexes

Cyclodextrins are interesting molecules due to their special structure. With their hydrophilic external surface and hydrophobic internal cavity, they can enclose lipophilic molecules such as flavonoids, improving their stability, solubility, and bioavailability. Cyclodextrins are widely used as excipients in different drug formulations, including oral, ophthalmic, dermal, and parenteral administration. The very first drugs containing parent cyclodextrin derivatives were administered parenterally and orally. The first parenteral injection containing prostaglandin E1 and α-cyclodextrin was marketed in 1976 in Japan [86]. Ten years later, the first tablet was marketed in Europe under the brand name Brexin, which is a β-cyclodextrin/piroxicam-containing medicine still in use today [87]. The number of CD-containing approved and marketed drugs is continuously increasing—from only 24 in 2004 to 129 products in 2022 [44]. Most of them were formulated with β-CD and its hydroxypropyl, sulfobutyl ether, random methyl, or sulfolipo derivatives. In oral formulations, the native/parent form is used, while in parenteral formulations, the sulfobutyl derivative is used. The hydroxypropyl derivative is used in both, as well as in ocular solutions [88].

The use of cyclodextrins can offer several additional benefits in drug formulation. Complexation can reduce the irritative effect of the active ingredient on mucous membranes or the skin, as cyclodextrin decreases the amount of free, irritation-causing active ingredient in the formulation. Taste improvement is also achievable: the bitter or unpleasant taste of flavonoids (e.g., naringin) can be masked through complexation, which is advantageous for orally administered preparations. Furthermore, some cyclodextrins can modulate the release of the active ingredient: with the appropriate composition and formulation of the complexes, slow, controlled release of the active ingredient can be achieved [88,89,90].

### 5.1. Formulations for Oral Application

Oral administration is one of the most common methods, as it is the easiest and most convenient for patients. During the formulation of tablets and oral solutions, mostly the parent β-CD and, to a smaller extent, HP-β-CD were used [88]. In the case of tablets, these excipients have many principal applications. They can be used to mask the bitter taste of active pharmaceutical ingredients. Sertraline is an antidepressant commonly used in the treatment of depression and anxiety. To enhance patient compliance and palatability, Kaushik et al. developed chewing gums loaded with a sertraline β-CD inclusion complex. The complexes were made by the kneading method, and the in vitro taste was investigated using E-Tongue. According to these results, the parent β-cyclodextrin was able to credibly mask the bitter taste of sertraline [91]. Another principal application is to modify or control the release of the active ingredient. Mostly, the hydrophobic and ionizable derivatives can be used in the formulation of sustained- and delayed-release tablets, and these excipients can be used to formulate noninvasive targeted active ingredient delivery systems [92]. The ability of parent and modified cyclodextrins was investigated in the formulation of inclusion complexes with dasatinib and their granulation and tableting. Tablets containing parent β-CD dasatinib inclusion complexes were identified with an improved dasatinib release profile, offering more controlled release, while HP-β-CD, due to its higher solubility, demonstrated a faster release [93]. Cyclodextrin can also act as a direct compression filler or disintegrating agent. Conceição et al. examined the ability of parent and HP-β-CD to act as a tablet filler for direct compression. Based on their results, both derivatives can be used as fillers; however, HP-β-CD showed better properties in both the physics of compression and drug release [94]. The principal application of these molecules is to increase the solubility, and therefore the bioavailability, of lipophilic drugs and to enhance their stability. Popielec and Loftsson described that CD complexation can hamper hydrolysis, oxidation, as well as enzyme-catalyzed degradation of the dissolved drug [95]. The effect of HP-β-CD on phenobarbital solubility and stability was investigated in oral pediatric solutions, and it was described that HP-β-CD solubilizes and stabilizes phenobarbital, setting the shelf life of this solution up to six months [96].

Some flavonoids are already used in dietary supplements or functional foods in the form of cyclodextrin complexes to improve their bioavailability. For example, there are *Ginkgo biloba* extracts embedded in γ-cyclodextrin, in which the flavonol components (quercetin, kaempferol derivatives) achieve higher plasma concentrations compared to traditional extracts. Overall, the main advantages of cyclodextrin complexes in oral formulations are faster dissolution, greater solubility, and stability in the gastrointestinal environment, resulting in consistent absorption curves and higher bioavailability [82,97].

### 5.2. Parenteral Administration Route

The parenteral route is another commonly used method of administration, besides oral administration, as it plays a significant role in emergency care by providing immediate action of the active ingredient after administration [90]. Cyclodextrins are mainly used as excipients in parenteral formulations, exploiting their solubility-enhancing properties. The main advantage of using these molecules is their ability to improve the solubility and stability of active ingredients, thereby enhancing their bioavailability, solubilization, and circulation time [13,98,99]. Furthermore, cyclodextrins can self-aggregate in aqueous solutions to form CD-based nanoparticles, which can create micelle-like nanoparticles or nanogels, depending on the production method and the polymers used [89]. In parenteral solutions, HP-β-CD and SBE-β-CD are primarily used due to their high water solubility and lower systemic toxicity [89]. Most preparations on the market contain anticancer drugs in an inclusion complex with cyclodextrin by itself, or this complex can be incorporated into liposomes and nanoparticles [90]. This approach can prolong the presence of the active ingredient in systemic circulation, reduce toxicity, and provide controlled, sustained, or targeted release of the drug [100]. HP-β-CD complex was prepared in a 1:1 ratio, and then these inclusion complexes were incorporated into liposomes. With this formulation, the water solubility, bioavailability, cellular uptake, and antitumor activity of kaempferol were enhanced compared to the free, uncomplexed active ingredient [101]. Quercetin-loaded liposomes were formulated and stabilized with HP-β-CD, improving the stability and bioavailability of the liposomes. Their results demonstrated enhanced skin permeability and penetration, and in vivo results showed improved treatment efficacy of psoriatic plaque compared to free quercetin [102].

Another option to improve the properties of inclusion complexes is the use of polymeric nanoparticles. Zheng et al. formulated catechin/cyclodextrin-encapsulated polymeric nanoparticles with PLGA to test their anti-virulence potential against pneumonia-causing human pathogens. According to their antibacterial tests, these nanoparticles showed a high loading capacity for catechin and cyclodextrin, functioning with synergistic effects as dual antibiotics against the human pathogens [103]. Pradhan et al. formulated chitosan nanoparticles containing quercetin-β-CD inclusion complexes and tested their neuroprotective potential against epilepsy. With this formulation, they were able to form successful inclusion complexes with cyclodextrin, reduce the crystallinity of quercetin, and achieve in vitro controlled active ingredient release compared to the free quercetin and the inclusion complex alone. Based on the in vivo studies, the nanoparticles, when administered orally, accumulated in the brain after 4 h, indicating improved bioavailability. This formulation was able to reduce seizure duration and frequency [104].

### 5.3. Topical Application

Besides oral and parenteral applications, cyclodextrins can be used for local treatment in ocular and dermal formulations. In dermal preparations, the parent beta-cyclodextrin is mostly used [88]. Cyclodextrin-based nanogels or hydrogels, as innovative drug delivery systems, can offer controlled active ingredient release in addition to enhanced solubility and improved bioavailability [105].

Due to their strong antioxidant and anti-inflammatory properties, flavonoids are promising active ingredients for topical skin treatments (e.g., anti-aging, inflammatory skin diseases, and wound healing support). However, their water insolubility and difficulty in penetrating the skin hinder their effective dermal application [106,107]. Cyclodextrin complexes offer advancements in this area. Firstly, complexed flavonoids are more soluble in water-based creams and gels, allowing formulations with higher active ingredient content without visible precipitation [108]. Secondly, cyclodextrin can deliver the guest molecule to the surface of the stratum corneum and, through equilibrium processes, transfer it to the skin, thereby facilitating penetration into deeper skin layers [109]. Additionally, the active ingredient in complex form is more protected against oxidative degradation, which is important for products like sunscreens or wound healing preparations [110]. Research has shown that the presence of cyclodextrin can enhance the local therapeutic effect of flavonoids. A notable example is the topical application of quercetin complexes: a transdermal gel containing quercetin and β-CD complex was developed and tested on rats for oxidative stress markers after skeletal muscle injury. The gel containing complexed quercetin significantly reduced lipid peroxidation and improved the activity of antioxidant enzymes (superoxide dismutase, catalase) in the injured muscle, indicating that the flavonoid effectively penetrated and exerted its protective effect. Interestingly, the effect achieved with the quercetin gel was practically identical to that achieved with ultrasound treatment (phonophoresis) applied by a therapist. This suggests that the cyclodextrin-solubilized flavonoid alone was able to provide a therapeutic level of effect in the tissues [111].

Another new approach is the use of cyclodextrin-based nanofibers on the skin. For example, a HP-β-CD/PVP nanofiber mesh containing myricetin was successfully produced, which, when applied to the skin as a patch, continuously released the flavonoid. This formulation, combining nanotechnology and complexation, significantly increased myricetin penetration into the skin and its photoprotective effect: it provided more effective protection in both in vitro keratinocyte models and in vivo UVB-induced skin damage compared to free myricetin, while showing good stability and skin compatibility [112].

Flavonoids embedded in cyclodextrin are often more resistant to environmental factors. Complexation can provide protection against heat, light, or oxidative degradation, thereby increasing the chemical stability and shelf life of the active ingredient. For example, it has been shown that encapsulating quercetin in cyclodextrin-based nanosponges significantly improves its light stability: complexed quercetin degrades much less under UV radiation compared to free quercetin. Additionally, quercetin in nanosponges exhibited stronger in vitro antioxidant activity than its free form, suggesting that cyclodextrin protects the molecule from inactivation and maintains its biological activity. Similarly, myricetin nanofibers produced in the presence of HP-β-CD showed increased photostability and thermal stability; these complexed nanofibers demonstrated enhanced antioxidant and photoprotective activity, effectively protecting the skin from UVB-induced damage. A more stable and effective active ingredient form can contribute to better therapeutic outcomes [113].

In ocular formulations, such as eye drops and in situ gels, hydroxypropyl- and sulfobutyl-beta-cyclodextrins are mostly used to enhance the solubility, bioavailability, and penetration of the drug. Gaetano et al. investigated the effects of rutin–SBE-β-CD inclusion complexes in solution on ocular infections. They found that the solubility of rutin was ten times higher in the inclusion complex than in the free rutin. Complexed rutin showed high activity against *Staphylococcus aureus*, while free rutin had no activity, indicating that cyclodextrin enhances the antibacterial and antibiofilm activity of rutin [42].

Overall, cyclodextrin complexes in topical formulations enhance the penetration and stability of the active ingredient, thereby increasing local efficacy.

### 5.4. Inhalatory Inclusion Complex Formulations

Cyclodextrin-complexed flavonoids can be used in inhalation forms for the treatment of respiratory diseases. In inhalable drug forms, such as aerosols and powder inhalation preparations, cyclodextrins facilitate the delivery of hydrophobic flavonoids to the lungs. CD complexes increase the water solubility of flavonoid active ingredients, allowing them to reach the airways in a stable powder or mist form.

An example is the complex of fisetin (a1n antioxidant flavonoid) and sulfobutyl ether-β-CD. A dry powder inhalation formulation was successfully prepared, which increased the solubility of fisetin more than a hundredfold, thereby facilitating the deep lung delivery of the active ingredient [114]. Similarly, quercetin–β-CD complexes have also been studied in inhalation powder form to prevent smoke-induced lung damage [40].

The inhalation delivery of CD-complexed flavonoids can be beneficial in chronic lung diseases (e.g., idiopathic pulmonary fibrosis or adjuvant therapy for lung cancer), as high local concentrations of the active ingredient can be achieved in the lungs while systemic side effects remain minimal [115]. Inhalatory flavonoid–CD formulations can thus open new therapeutic avenues for the treatment of respiratory diseases, combining the beneficial effects of natural flavonoids with modern carrier technology.

### 5.5. Nanoformulation

**Figure 7:**
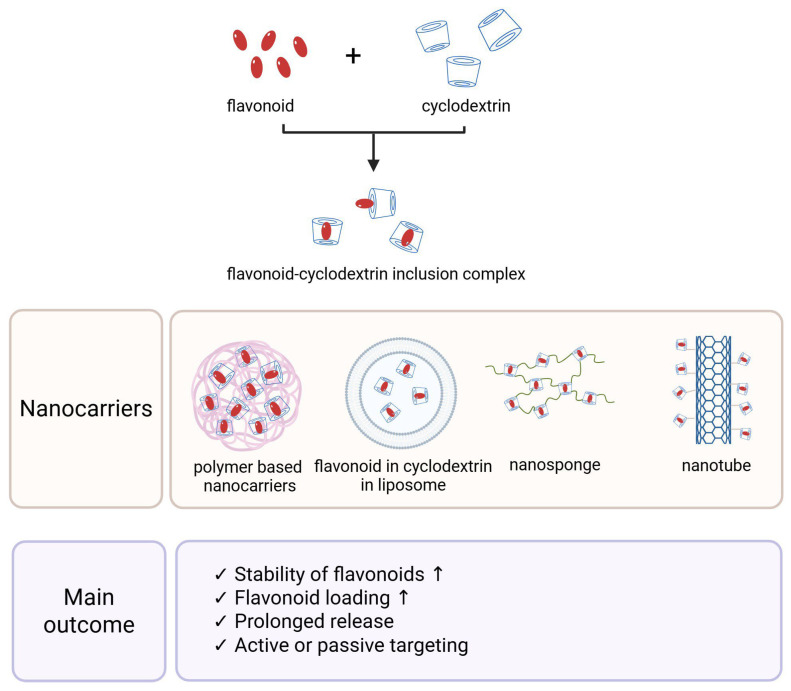
Nanoformulation opportunities of flavonoid–cyclodextrin complexes.

Various cyclodextrins (β-CD, γ-CD, and especially well-soluble derivatives like HP-β-CD) can encapsulate flavonoid molecules in the form of inclusion complexes, which, when incorporated into liposomes, create new-generation delivery systems (see Figure 7). These systems improve the solubility and stability of flavonoids, increase the deliverable doses, and enable controlled or targeted release [77,116].

CD–flavonoid liposomes are also promising in targeted active ingredient delivery. The encapsulation of flavonoid complexes into liposomes aids passive accumulation, as the longer circulation time and stability allow more active ingredients to reach the target tissue. Additionally, active targeting can be achieved by attaching ligands (e.g., tumor cell-specific antibodies or folate groups) to the surface of the liposomes, and the carrier can be directed to the desired cell surface receptors [117]. Several research groups have studied quercetin liposomal CD complexes. Basaran and colleagues demonstrated that the HP-β-CD-complexed quercetin liposomal formulation selectively inhibits tumor cell proliferation: in vitro, the quercetin complex exhibited stronger antiproliferative effects on human breast and lung cancer cell lines (MDA-MB-231, A549) compared to healthy fibroblasts, while the rutin complex mainly inhibited breast cancer cells. Moreover, the combined complex of quercetin and rutin synergistically increased tumor cell cytotoxicity [77]. The effect of HP-β-CD-stabilized quercetin-liposome gel was investigated in a psoriasis mouse model: the topically applied system improved the healing of psoriasis plaques, reduced skin thickening, and significantly decreased the levels of inflammatory cytokines (TNF-α, IL-17A, IL-1β) in the treated tissue compared to untreated and free quercetin-treated controls [102]. This suggests that CD-coordinated liposomes are not only stable but also enhance anti-inflammatory effects at the appropriate target site.

In recent years, nanoformulations have emerged in which flavonoid–CD complexes are further developed into nanotechnological carriers. These include cyclodextrin-based nanosponges, nanogels, nanoemulsions, or CD-functionalized nanoparticles. These systems combine the advantages of cyclodextrin complexation with the characteristics of nanotechnology: the active ingredient becomes both water-soluble and bound to a nano-sized carrier, which can be more precisely delivered to the desired location in the body [118].

A typical example is silymarin (a flavonoid extract from milk thistle) embedded in β-CD-based nanosponges, which showed increased antitumor effects on melanoma cell lines compared to free silymarin [119,120]. The nanosponge formulation improved the stability of the active ingredient and its sustained release, resulting in stronger antioxidant, anti-inflammatory, and antitumor activities [119,120].

**Figure 8:**
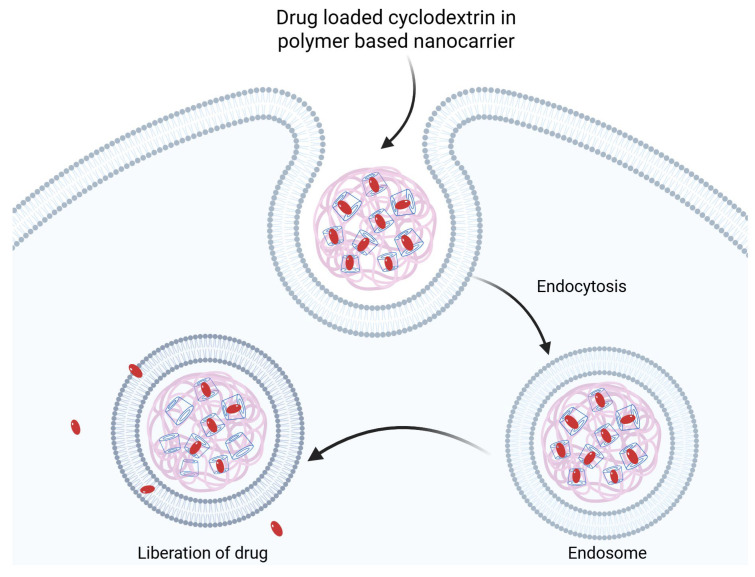
Liberation of encapsulated flavonoids from nanocarrier containing endosome.

Further research involved loading quercitrin flavonoid into a cyclodextrin nanoparticle network, achieving controlled release of the active ingredient and better bioavailability in targeted tissues [121]. Nano-sized CD carriers (e.g., nanosponges) are particularly promising in anticancer therapies and other diseases, as nanoparticles easily enter cells, and cyclodextrin stably carries the flavonoid to the target site. The mechanism of nanoparticle entry into the target cell and release of the guest molecule is shown in Figure 8.

The chemical structure, low octanol/water partition coefficient, high molecular weight, and hydrophilic nature of cyclodextrins predict that they do not penetrate biological membranes, including the membranes of endosomes and lysosomes. Only the dissociated, free form of the complexed molecules permeates the lipid membranes [122].

There is a large energy barrier for β-cyclodextrins and their complexes to penetrate biological membranes [123].

The escape of cyclodextrins, polymers, or nanocarriers from endosomes is generally believed to occur only upon disruption of the endosomal or lysosomal membrane. This disruption mechanism has been reported for certain polymers, such as polyethylenimine (PEI), which can buffer protons in lysosomes, leading to osmotic swelling, membrane rupture, or destabilization—a phenomenon commonly referred to as the “proton sponge effect”. However, the validity of this mechanism remains debated [124]. To the best of our knowledge, this mechanism has not yet been described for cyclodextrins.

Overall, nanoformulations based on flavonoid–cyclodextrin complexes are among the innovative delivery systems of the future, providing better solubility, stability, and targeted delivery for natural flavonoids.

## 6. Biological Effect and Therapeutic Applications

### 6.1. Neuroprotective Effects of Flavonoids and CD Complexes

Hesperetin is an aglycone form of hesperidin, which is a flavonone. It derives from citrus and is famous for its neuroprotective, anti-inflammatory, and antioxidant effects [125,126]. Hesperetin penetrates the blood–brain barrier and alleviates the inflammation and oxidative stress [127,128,129]. Moreover, it can control the endoplasmic reticulum stress and maintain a good mitochondrial function [130,131], so it is a promising model in Alzheimer’s disease. Hesperetin also significantly keeps down the production of NO, induced by LPS in cultured microglial cells [132].

Quercetin has a strong neuroprotective effect as an antioxidant because it scavenges ROS, which protects the neurons from oxidative stress-related damage. Quercetin reduces the levels of pro-inflammatory cytokines like IL-6, TNF-α, and IL-1β. Preclinical studies demonstrated the effects in neurodegenerative models, including protection against ß-amyloid-induced neuronal damage. Quercetin has shown positive therapeutic effects in both in vivo and in vitro as a treatment for Alzheimer’s and Parkinson’s diseases [133,134].

In experimental models of Alzheimer’s disease, quercetin has been demonstrated to have the ability to attenuate chronic neuroinflammation. Specifically, quercetin also reduces the expression of pro-inflammatory mediators like nuclear factor kappa B (NF-κB) or inducible nitric oxide synthase (iNOS), both of which are critically involved in sustaining inflammatory cascades in the brain [133]. In microglial models, quercetin attenuates NO production in response to LPS stimulation, which is mechanistically linked to the downregulation of extracellular signal-regulated kinases (ERK, p38 MAPK) and the inhibition of NF-κB signaling [135]. The neuroprotective effects of quercetin have been further verified in preclinical models of Parkinson’s disease, particularly in the well-established 6-hydroxydopamine (6-OHDA)-induced lesion model [134,136]. In this experiment, quercetin was administered at 10 and 25 mg/kg doses, significantly alleviating cognitive impairments commonly observed in PD.

The neuroprotective actions of quercetin exemplify the broader therapeutic promise of flavonoids in age-related neurodegenerative conditions, where both oxidative stress and inflammation are intricately involved.

In a recent study they compared oral and intranasal quercetin–cyclodextrin powders in a rat model [137]. Intranasal quercetin powders administered in the form of HP-β-CD or Me-β-CD complexes mixed with mannose/lecithin microparticles achieved significantly higher brain concentrations than the orally administered solution (negligible amounts of compound were delivered to the brain compared to oral). This suggests that intranasally or orally delivered (nose-to-brain) formulations of such complexes may be suitable for preventing or treating neurodegenerative diseases. In particular, it is promising that the cyclodextrin-complexed quercetin formulation was well delivered to the circulation and brain after oral administration. Pharmacokinetic data from intranasal and oral administration showed that the intranasal regimen resulted in significantly higher plasma and brain levels, whereas these were barely measurable in the oral administration [137]. In another study, they investigated the quercetin-loaded nanoparticles containing HP-β-CD for oral administration for at least 2 months in senescence-accelerated mouse prone 8 (SAMP8) mice. They tested the motor activity and memory in two groups: quercetin alone and in nanoparticles. At the dose of 25 mg/kg, quercetin loaded in nanoparticles resulted in improved memory and cognition. The exact dosage of quercetin did not have any significant effect alone [138]. These results support further research and application of flavonoid–cyclodextrin complexes in the prevention and treatment of neurodegenerative diseases.

Luteolin, a flavone commonly found in herbs and vegetables, has demonstrated significant neuroprotection and anti-inflammatory effects. It keeps down the expression of inflammatory mediators such as IL-6, COX-2, and iNOS in activated microglia cells, as well as astrocytes [139]. Luteolin has an effect by inhibiting signaling pathways like NF-κB, MAPK, and JNK [140,141].

Catechins, a subgroup of flavanols abundant in green tea, have demonstrated potent antioxidant and anti-inflammatory effects in the central nervous system. Epigallocatechin gallate (EGCG), a major catechin abundant in green tea, protects microglial and neuronal cells from oxidative stress-induced damage by inhibiting DNA damage, lipid peroxidation, and inflammatory signalling [142,143,144]. EGCG downregulated inflammation as a result of inhibiting the COX-2 and iNOS expressions and the production of inflammatory cytokines in cultured astrocytes and in the mouse brain [145]. Catechins have also been shown to suppress the activation of the NF-κB pathway, which is involved in microglia-mediated neurotoxicity [141].

Anthocyanins, a class of anthocyanidins with glycosylated sugar residues, are highly effective in neuroinflammation and oxidative stress. Studies demonstrate that anthocyanins can suppress the activation of microglia and astrocytes, reduce NF-κB signalling, and decrease levels of pro-inflammatory cytokines [146]. Moreover, anthocyanins enhance neuron–microglia communication by modulating CX3CL1/CX3CR1 signalling and supporting synaptic integrity [147]. Anthocyanins are able to reduce neuroinflammation, which has been well-described in aging models and in demyelination-related neurodegeneration [148].

Genistein, an isoflavone found in soy products, acts as a phytoestrogen and displays notable neuroprotective activity. It has anti-inflammatory effects by inhibiting the expression of IL-6, MCP-1, IRF-1, and STAT1 in LPS-stimulated microglial cells [149]. Genistein also has anti-apoptotic properties, protecting neurons against HIV-Tat-induced cytotoxicity via estrogen receptor-mediated signalling [150]. These mechanisms collectively support genistein’s therapeutic potential in neuroinflammatory conditions and neurodegenerative diseases.

### 6.2. Anti-Inflammatory Effects of Flavonoids and CD Complexes

Flavonoids exert anti-inflammatory effects through multiple pathways, including suppressing important regulatory enzymes and transcription factors involved in modulating pro-inflammatory mediators. In addition to their anti-inflammatory activity, flavonoids possess strong antioxidant capacity, enabling them to neutralize reactive oxygen species and limit their generation. As a result, flavonoids influence various immune cells and pathways central to the inflammatory response [151].

A major target of flavonoids is the NF-κB signalling pathway, which controls the level of inflammatory genes such as TNF-α, IL-1β, and IL-6 [152]. Under inflammatory stimuli, IκB is phosphorylated and degraded, enabling NF-κB to enter the nucleus and promote pro-inflammatory gene expression [152,153]. Flavonoids can counteract this process by preventing IκB degradation and NF-κB nuclear translocation. They also modulate Th2 immune responses by affecting transcription factors like GATA-3 and STAT-6 [139,154].

Both in vivo and in vitro studies have shown that quercetin effectively reduces inflammation by inhibiting COX-2, LOX, and iNOS activity, lowering cytokine production, and suppressing NF-κB activation [155,156,157].

Meng et al. investigated the anti-inflammatory effects of encapsulated hesperetin (HST) by β-CD. Caco-2–THP-1 cell co-culture was treated, and the inflammation was stimulated with LPS. As a result of the LPS treatment, the levels of the pro-inflammatory cytokines and inflammation-related genes were higher compared to the untreated ones. HST-CD complex was effective in the suppression of IL-8 cytokine level; however, it did not decrease the Myd88, NF-κB, or COX-2 levels. The reason can be because of the hydrophilic outer surface of cyclodextrin, which improved the solubility of HST-CD complex, the medium matrix could influence the stability of this complex, resulting in the release of hesperetin [158].

The study used a CCl_4_-induced liver fibrosis model in mice. This is a well-established animal model that induces chronic inflammation and progressive fibrotic lesions in the liver, similar to human liver cirrhosis. Chrysin is a natural flavonoid (e.g., found in royal jelly) with known anti-inflammatory and antioxidant effects. However, due to its poor water solubility and bioavailability, it is not very effective on its own at a therapeutic level. HP-β-CD and RAMEB (randomized methylated β-cyclodextrin) are used to form soluble, stable complexes of chrysin, which increase the bioavailability and cellular uptake of the chrysin. It significantly reduced levels of inflammatory cytokines, including TNF-α and IL-1β. Immunohistochemical staining and qPCR analysis showed that the application of the complexes inhibited activation of NF-κB signalling. TGF-β1 and Smad3 expression were significantly downregulated, the major pro-fibrotic signalling pathway in hepatocyte activation. The expression of α-SMA (alpha-smooth muscle actin) and collagen I—markers of fibrosis—is also reduced. The histological structure of the liver was significantly improved (reduced fibrosis, regenerated hepatocytes). Serum transaminase levels (ALT, AST) returned to near normal values, indicating improved liver function. Overall, the chrysin-HP-β-CD and chrysin-RAMEB complexes effectively reduced inflammation and fibrotic transformation in the liver through a clear mechanistic effect (NF-κB and TGF-β/Smad inhibition). These results support the potential of flavonoid–cyclodextrin systems also in the therapy of chronic liver diseases [159].

### 6.3. Cardiovascular Protection of Flavonoids and CD Complexes

In the last few years, polyphenolic compounds (particularly anthocyanins) have gained a higher potential in the prevention and treatment of chronic diseases, including cardiovascular disease (CVD) [160]. A growing body of evidence supports the cardioprotective effects of anthocyanins, which include attenuation of inflammation, improvement of endothelial function, and enhancement of nitric oxide (NO) bioavailability [161,162].

A placebo-controlled trial was conducted in a randomized manner, involving 120 participants who received either 320 mg/day of purified anthocyanins from black currant (Ribes nigrum) or bilberry (Vaccinium myrtillus) or a placebo for 12 weeks. Compared to the placebo group, anthocyanin supplementation significantly enhanced HDL-cholesterol and cholesterol efflux capacity, while reducing LDL-cholesterol and cholesteryl ester transfer protein (CETP) levels [163]. The authors attributed these effects to CETP inhibition. In subsequent studies using the same extract, the group also reported increased HDL-associated paraoxonase 1 (PON1) activity, plasma cGMP, and improved flow-mediated dilation, suggesting enhanced endothelial function via the NO–cGMP pathway [164,165].

Several cross-sectional studies have reported inverse associations between higher anthocyanin intake and various inflammatory biomarkers, including C-reactive protein (CRP), interleukin-18 (IL-18), as well as a composite inflammation score incorporating multiple cytokines, acute-phase reactants, and oxidative stress markers [166].

The association between habitual flavonoid intake and cardiovascular risk was investigated in 43,880 men aged 32–81 years over a 24-year follow-up. Higher anthocyanin consumption—up to 613 mg/day—was associated with a 14% reduction in the risk of nonfatal myocardial infarction [167]. Similar associations had previously been reported in female cohorts [168,169].

It is well established that apoptosis is a key factor in the development of heart failure and myocardial diseases. Hesperetin exhibits anti-apoptotic properties, making it a promising candidate for treating these conditions. Its anti-apoptotic effect was confirmed in H9C2 cardiomyocytes following LPS stimulation. The findings clearly indicated that Hesperetin significantly reduced apoptosis in these cells via the apoptotic pathway [170].

Quercetin, a bioactive flavonoid found in various vegetables and dietary sources, has garnered considerable attention for its cardioprotective properties [171,172]. It exerts beneficial effects on cardiovascular health, including inhibiting low-density lipoprotein (LDL) oxidation, endothelium-independent vasodilation, and the downregulation of adhesion molecules and other pro-inflammatory biomarkers. Additionally, quercetin supports nitric oxide bioavailability and endothelial function under oxidative stress conditions, helps prevent oxidative and inflammatory neuronal damage, and displays antiplatelet aggregation activity. Furthermore, several studies have highlighted quercetin’s role in lowering blood pressure, alongside its potent antioxidant capacity and cardioprotective effects [173].

Luteolin demonstrates significant cardioprotective effects mediated through intricate signal transduction pathways and molecular targets. Notably, a high dietary intake of luteolin has been related to a reduced risk of acute myocardial infarction [174,175]. Its cardiovascular benefits are primarily attributed to the attenuation of myocardial apoptosis and reduced infarct size [176].

In a very recent study, they developed the hesperetin-7-glucoside–β-cyclodextrin inclusion complex (HCD). As compared to hesperetin alone, the complex presents better solubility and bioavailability. They administered 300 mg/day HCD for 12 weeks, and they found that in the case of HCD-treated patients, a significant improvement in endothelial dysfunction was observed [177].

Another flavanone was also studied, dioclein. In the study, a 1:1 inclusion complex was formed between dioclein and β-CD, which increased the water solubility of the flavonoid by 44%. Intraperitoneal administration of 2.5 mg/kg of the complex caused a significant and sustained reduction in systolic blood pressure compared to the free form. When administered orally, free dioclein (10 mg/kg) did not reduce blood pressure, whereas the dioclein-β-CD complex did, which clearly supports its oral hypotensive effect [178].

Catechins found in green tea, such as epigallocatechin gallate (EGCG), have demonstrated protective effects against cardiovascular diseases These polyphenolic compounds exert antioxidant activity by scavenge ROS, chelate metal ions, and enhance the activity of endogenous antioxidant enzymes like catalase, superoxide dismutase (SOD) and glutathione peroxidase, thereby mitigate oxidative stress which is a contributor to cardiovascular pathology [179]. In experimental models, catechins have been shown to improve vascular endothelial function, reduce blood pressure, and prevent the growth of vascular smooth muscle cells, as well as reduce lipid peroxidation and inflammatory responses by downregulating cytokines like TNF-α and adhesion molecules such as ICAM-1 [180,181]. Furthermore, catechins help restore lipid metabolism and prevent the accumulation of cholesterol in arterial walls, contributing to the prevention of atherosclerosis and myocardial damage during ischemic events [182].

In the last twenty years, there has been a growing scientific interest in anthocyanins (polyphenolic compounds predominantly found in berries and berry-based products) due to their well-documented cardioprotective properties associated with regular intake of anthocyanin-rich foods.

In a 16-year prospective cohort study of 34,489 healthy postmenopausal women aged 55–69, higher anthocyanin intake was related to a 12% reduction in the risk of coronary heart disease and a 9% decrease in cardiovascular disease-related mortality [169]. A recent meta-analysis of 45 RCTs found that berry and purified anthocyanin supplementation (2.2–1230 mg/day) significantly increased HDL-cholesterol and reduced LDL-cholesterol, triglycerides, blood pressure, and inflammatory markers (CRP, TNF-α). Subgroup analyses indicated stronger effects in overweight individuals, adults ≥50 years, and those at elevated cardiovascular risk [183].

It has been revealed that Genistein lowers serum cholesterol, inhibits tyrosine kinase, and enhances vascular reactivity, possibly due to its antioxidant activity [184,185]. Moreover, soy isoflavones and their glycosides were related to lower cardiovascular diseases [186].

## 7. Conclusions

The poor physicochemical properties of flavonoids can be significantly improved by complexing with cyclodextrins, providing opportunities for the implementation of different formulations. Their antioxidant properties make them widely considered therapeutically, which is significantly enhanced by their nanoformulations. These innovative nanocarrier preparations not only expand the therapeutic potential of flavonoids—in areas such as neurodegenerative diseases, inflammation, and cancer—but also open new directions in the development of functional foods, topical treatments, and inhalation therapies. One of the greatest health challenges of our time is to increase the effectiveness of preventive therapies, which can be achieved through the beneficial interaction of flavonoids and cyclodextrin derivatives. Overall, cyclodextrin-based nanocarriers enable more efficient, stable, and targeted application of natural antioxidants, thus becoming key elements of next-generation delivery systems.

## Abbreviations

The following abbreviations are used in this manuscript:

Akt	Protein Kinase B
CD	Cyclodextrin
CETP	Cholesteryl Ester Transfer Protein
cGMP	Cyclic Guanosine Monophosphate
COX-2	Cyclooxygenase-2
CRP	C-Reactive Protein
DM-β-CD	Dimethyl-Β-CD
DNA	Deoxyribonucleic Acid
DPPH	2,2-Difenil-1-Pikrilhidrazil
ERK	Extracellular Signal-Regulated Kinases
ESI-MS	Electrospray Ionization Mass Spectrometry
HP-β-CD	Hydroxypropyl-Βeta-Cyclodextrin
iNOS	Inducible Nitric Oxide Synthase (Inos
LPS	Lipopolysaccharide
Me-β-CD	Methylated Βeta-CD
MM PBSA	Molecular Mechanics Poisson–Boltzmann Surface Area
Myd88	Myeloid Differentiation Primary Response 88 Protein
NF-κB	Nuclear Factor-Kappa B
PI3K	Phosphatidylinositol 3-Kinase
PLGA	Poly(Lactic-Co-Glycolic Acid)
PON1	Paraoxonase 1
PVP	Polyvinylpyrrolidone
RAMEB	Random Methylated-Β-CD
ROS	Reactive Oxygen Species
SBE-β-CD	Sulfobutyl Ether-Βeta-Cyclodextrin
SOD	Superoxide Dismutase
STAT-6	Signal Transducer and Activator of Transcription 6
XIAP	X-Linked Inhibitor of Apoptosis Protein
